# Intracisternal delivery of PEG-coated gold nanoparticles results in high brain penetrance and long-lasting stability

**DOI:** 10.1186/s12951-019-0481-3

**Published:** 2019-04-03

**Authors:** Antonello Spinelli, Maria Girelli, Daniela Arosio, Laura Polito, Paola Podini, Gianvito Martino, Pierfausto Seneci, Luca Muzio, Andrea Menegon

**Affiliations:** 1 Experimental Imaging Centre, San Raffaele Scientific Institute, 20132 Milan, Italy; 2 Neuroimmunology Unit, Division of Neuroscience, Institute of Experimental Neurology (INSPE), San Raffaele Scientific Institute, 20132 Milan, Italy; 3 Institute of Molecular Science and Technologies (ISTM), CNR, Via C. Golgi 19, 20133 Milan, Italy; 4 Neuropathology Unit, Division of Neuroscience, Institute of Experimental Neurology (INSPE), San Raffaele Scientific Institute, 20132 Milan, Italy; 5 Chemistry Department, Università degli Studi di Milano, Via Venezian 21, 20133 Milan, Italy

**Keywords:** Gold nanoparticles, Intra cisterna magna, In vivo analysis

## Abstract

**Background:**

The increasing use of gold nanoparticles (AuNPs) in the field of neuroscience instilled hope for their rapid translation to the clinical practice. AuNPs can be engineered to carry therapeutics or diagnostics in the diseased brain, possibly providing greater cell specificity and low toxicity. Although there is a general enthusiasm for these tools, we are in early stages of their development. Overall, their brain penetrance, stability and cell specificity are critical issues that must be addressed to drive AuNPs to the clinic.

**Results:**

We studied the kinetic, distribution and stability of PEG-coated AuNPs in mice receiving a single injection into the cisterna magna of the 4th ventricle. AuNPs were conjugated with the fluorescent tag Cy5.5 (Cy5.5-AuNPs) to track their in vivo distribution. Fluorescence levels from such particles were detected in mice for weeks. In situ analysis of brains by immunofluorescence and electron microscopy revealed that Cy5.5-AuNPs penetrated the brain parenchyma, spreading in the CNS parenchyma beneath the 4th ventricle. Cy5.5-AuNPs were preferentially found in neurons, although a subset of resting microglia also entrapped these particles.

**Conclusions:**

Our results suggest that the ICM route for delivering gold particles allows the targeting of neurons. This approach might be pursued to carry therapeutics or diagnostics inside a diseased brain with a surgical procedure that is largely used in gene therapy approaches. Furthermore, this approach could be used for radiotherapy, enhancing the agent’s efficacy to kill brain cancer cells.

**Electronic supplementary material:**

The online version of this article (10.1186/s12951-019-0481-3) contains supplementary material, which is available to authorized users.

## Background

A wide interest in the field of nanomedicine involves structurally different nanovectors, and in particular functionalized nanoparticles. Given their excellent biocompatibility and stability, nanoparticles are used to deliver drugs, genes, fluorescent labels, imaging agents, and enhancing radiotherapy tools. More recently, they were proposed as antiviral agents [1, 2]. Accordingly, more than 200 nano-objects targeted toward nanomedicine are currently approved for clinical trials in patients, while a larger number is engaged in pre-clinical studies [3].

Despite encouraging results indicate that nanovectors are reliable tools to treat a considerable number of pathologies, their use in disorders of central nervous system (CNS) is hampered by some limitations. A major challenge for their usage in CNS is their limited ability to cross the blood brain barrier (BBB), which often prevents to attain effective pharmacokinetic levels of therapeutics in the brain. Recent advances led to the generation of improved nanoparticles showing increased CNS penetration [4], therefore opening new avenues in basic and clinical neuroscience [5]. Among such particles, metallic nanoparticles are extremely attractive tools for their biophysical characteristics [6]. Gold nanoparticles (AuNPs) display unique optical and electronic properties to be considered reliable tools for drug/gene delivery, biomedical imaging [7], photothermal and microwave therapies [8]. AuNPs can be easily and uniformly functionalized with different chemical groups, allowing these particles to target specific locations in different organs as well as to reduce toxicity [9, 10]. AuNPs display size-dependent kinetics and distribution when injected in tissues [11, 12]. The intravenous injection of AuNPs results in accumulation of particles in several organs, including the brain. Interestingly, particles with sizes in the range of 15–50 nm cross the BBB better than particles with bigger diameters [12, 13]. The surface functionalization has a great impact on pharmacokinetic properties of AuNPs [4]. For example, transferrin-coated nanoparticles enter the brain through transcytosis and are more BBB-penetrant than other types of functionalized AuNPs [14]. Polyethylene glycol (PEG) coating of AuNPs increases their solubility and reduces toxicity [15]. Indeed, in a model of spinal cord contusion the local injection of PEG-coated AuNPs improved functional recovery and attenuated demyelination [16]. Insulin coated AuNPs crossed the BBB, reaching the brain with concentrations that are around 5% of the total injected dose [17]. However, 2 days after the delivery, the concentration of particles in the brain drops to values around 1–2% of the injected dose [13]. Imaging studies further corroborate these observations, showing that once AuNPs get into the brain, they rapidly disappear [17–21]. Intra-carotid injection of AuNPs increases the penetrance of particles in the brain, although their diffusion in the parenchyma is limited to few microns from the visible vessels [22]. Repeated treatments can increase the penetrating amount of AuNPs in the brain [6, 17, 23], although an uncontrolled accumulation of AuNPs in neurons could create some adverse effects, such as alterations of the firing properties of neurons [24]. Therefore, we must take in consideration such aspect if we plan therapeutic strategies for chronic diseases/applications.

The use of intra-parenchymal injections or the implantation of catheters, both by-passing the BBB, have the great limit of requiring invasive surgical procedures. Likely, both approaches can trigger undesired local inflammatory responses [25, 26]. A strategy to increase the penetrance and the permanence of appropriate concentrations of AuNPs in the CNS, that avoids the use of serial injections, requires alternative routes of administrations. In a model of focal lesion, such as the spinal cord injury, PEG-AuNPs injected in two sites that flanked the lesion epicenter efficiently supported the functional recovery of mice [16]. In spite of this noteworthy result, we can speculate that such procedure will find some limitations in diseases featuring large or disseminated brain lesions, such as multiple sclerosis (MS). Among alternative approaches to delivery AuNPs, the intrathecal route offers several advantages and it has been used to delivery drugs, as well as to infuse the brain with anesthetics, trophic factors and antibodies, although it has never been used to deliver nanoparticles [27, 28]. Experiments of gene delivery in mice clearly demonstrated that the intra cisterna magna (ICM) route is safe [29–31]. Indeed, gene therapy experiments showed that ICM injection of lentivirus leads viruses to diffuse consistently in all cerebrospinal fluid (CSF) spaces, infecting both choroidal and ependymal cells [29].

Growing evidence from several studies suggests that non-invasive imaging procedures to track the bio-distribution of AuNPs at different time points are excellent tools to accomplish the goal of using AuNPs in a clinical setting. The in vivo near infrared optical imaging of fluorescent-coated AuNPs is a relatively novel fluorescence imaging (FLI) approach that overcomes some previous limits of in vivo imaging, offering an excellent resolution and, above all, a limited invasiveness [17].

In this study, we characterized mice receiving ICM delivery of PEG functionalized and Cy5.5 labelled AuNPs (Cy5.5-AuNPs). We observed robust accumulation of Cy5.5-AuNPs in the CNS of mice, receiving a single injection of particles. Injected mice did not show signs of distress or pain. Remarkably, in vivo imaging showed detectable signals for more than 20 days. Confocal and electron microscopy analyses revealed that Cy5.5-AuNPs internalization in scattered neurons along the CNS parenchyma with some of them located several hundred microns far from the ependymal layer.

## Results

### Synthesis and characterization of Cy5.5-AuNPs

**Fig. 1:**
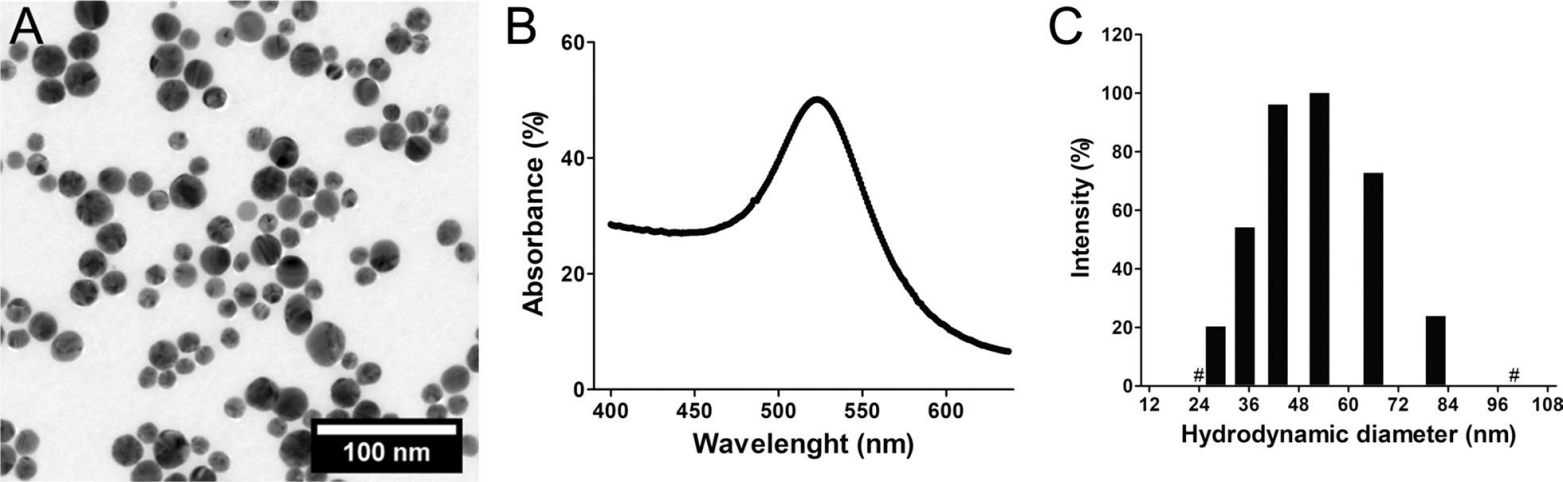
Characterization of AuNPs. **A** TEM image of AuNPs. The AuNPs have a spherical shape with a diameter (mean ± S.D.) of 18.03 ± 4.93 nm. **B** UV–vis spectrum of AuNPs. The maximum absorbance of the plasmonic peak is at 523 nm. **C** Hydrodynamic diameter (nm) distribution of Cy5.5-AuNPs, obtained by dynamic light scattering (DLS) measurements. The intensity-mediated measure gives a mean hydrodynamic diameter of 50.92 nm

We recently developed a new method based on a Turkevich-modified ‘one-pot’ strategy to obtain large batches of functionalized AuNPs, that avoids the purification of intermediate citrate-stabilized nanoparticles [32]. PEG coating guarantees the indispensable colloidal stability and water dispersibility, making AuNPs undetectable by macrophages [33]. Taking advantage from this functionalization, we conjugated PEG-AuNPS with the near infrared fluorescent dye Cy5.5 (excitation/emission: 674/710 nm, having an ideal spectrum for in vivo imaging and limiting tissue auto-fluorescence). Purified Cy5.5-AuNPs were stable for months when kept at 4 °C, without any trace of nanoparticles aggregation or Cy5.5. dye degradation as detected by DLS and fluorescence measurements. The obtained spherical Cy5.5-AuNPs showed a size distribution centered at 18.03 ± 4.9 nm as determined by transmission electron microscopy (TEM) analysis (Fig. 1A). They showed the typical UV–vis spectrum with the maximum plasmonic absorption band at 523 nm (Fig. 1B). The presence of high molecular weight PEG coating the metal surface is confirmed by the determination of a hydrodynamic diameter value of 50.92 nm (Fig. 1C and Additional file 1: Figure S1) [34].

### In vitro characterization of Cy5.5-AuNPs toxicity

**Fig. 2:**
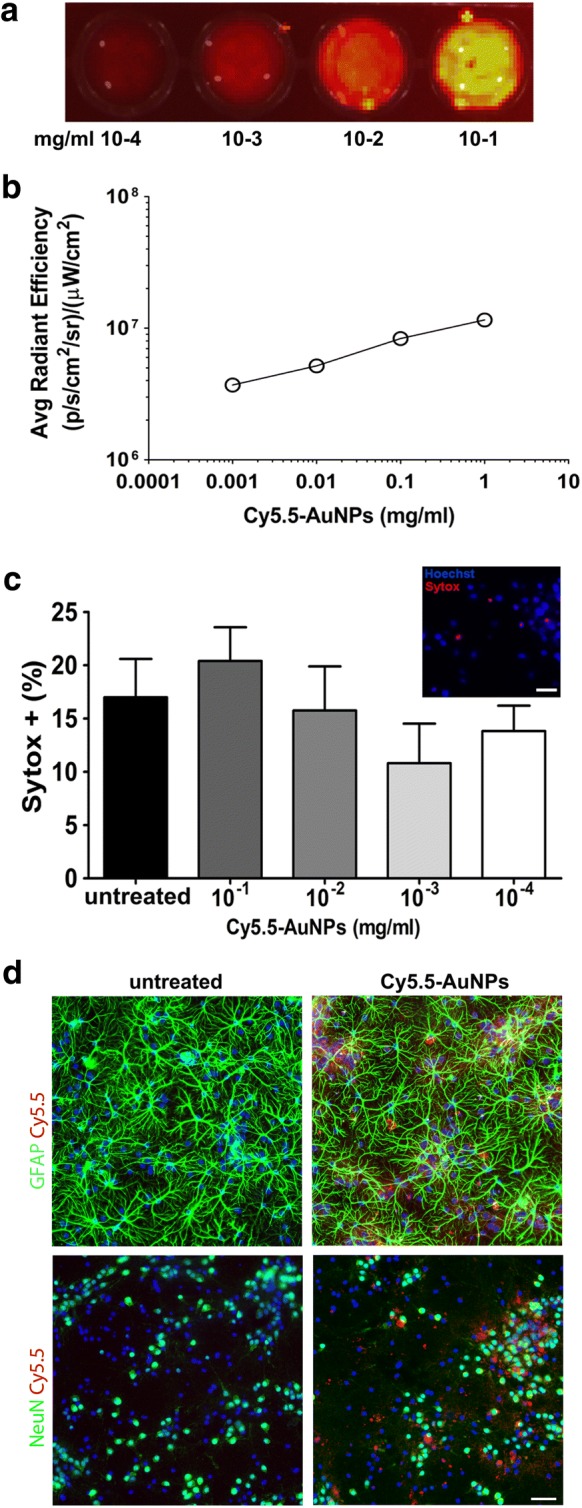
Cy5.5AuNPs do not elicit cytotoxicity in primary neuronal cultures. **a** Four representative plates of a 96 wells-plate containing 200 μl of Cy5.5-AuNPs at four different concentrations that range from 1 to 0.001 mg/ml. Images were acquired using the 675/720 filters and averaged radiant efficiencies were measured using a circular ROI superimposed on each well. Histogram of **b** shows the linearity between the radiant efficiency and AuNPs concentration. **c** Percentages (± S.D.) of dying hippocampal neurons in cultures treated with AuNPs. Percentages (± S.D.) of dying cells, scored with Sytox, are shown on **c**. Inset shows a representative picture derived from cultures receiving AuNPs for 24 h, n = 4 independent cultures. **d** Au-NPs distribution in hippocampal cultures receiving 0.1 mg/ml of Cy5.5-NPs for 24 h. Cultures were labelled for GFAP to identify astrocytes (upper row **d**) and NeuN to identify neurons (lower row **d**). Scale bar 15 µm for **c** and 40 µM for **d**

We assayed the cytotoxicity of Cy5.5-AuNPs on primary neuronal cultures established from the hippocampus of E17.5 murine embryos. Neurons, cultured 15 days in vitro, exhibited the typical arborization of mature cells. At this time point, they usually display spontaneous neuronal activity organized in functional networks [35, 36]. We used FLI to establish fluorescence levels of a range of Cy5.5-AuNPs concentrations. We imaged plates containing increasing concentrations of particles and using 675/720 filters we established a correlation between fluorescence levels and AuNPs concentrations (Fig. 2a, b). We next incubated neurons with increasing amount of Cy5.5-AuNPs to measure rates of cell death. Twenty-four hours after seeding neurons with particles, we evaluated percentages of dying neurons scoring Sytox^+^ cells (Fig. 2c). We observed a slight increase of cell death rates in neurons receiving the highest concentration of Cy5.5-AuNPs, although these levels did not statistically differ from values recorded in untreated neurons (Fig. 2c). However, lower concentrations of Cy5.5-AuNPs did percentages of Sytox^+^ cells that not exceed rates measured in untreated cultures (Fig. 2c). We next fixed and labelled cells for NeuN and GFAP, which are markers of neurons and astrocytes respectively. We did not observe unspecific Cy5.5 signals in untreated cells (Fig. 2d, right column). Cultures receiving Cy5.5-AuNPs showed Cy5.5 fluorescence in regions containing clusters of NeuN^+^ cells. While the localization of Cy5.5 signals appeared uncorrelated with the distribution of GFAP^+^ cells, (Fig. 2d left column). Altogether, these results suggests a general low cytotoxicity of PEG-coated Au particles for cultured hippocampal neurons [37].

### In vivo delivery of Cy5.5-AuNPs

We next administered AuNPs to adult mice, establishing their pharmacokinetic distribution in the brain. We initially performed a single s injection of Cy5.5-AuNPs in the tail vein of mice. We used an equal amount of soluble Cy5.5 to establish control mice. We recorded FLI 5 days after the delivery of Cy5.5-AuNPs. We calculated the fluorescence ratio (FR) in each animal (see “Methods” sections for details). However, fluorescence levels measured in both groups did not exceed the background levels, and did not allow a robust calculation of FR levels (Additional file 2: Figure S2A). We next examined Cy5.5 fluorescence in the cortical wall of these mice using the NeuN marker to identify neurons. However, levels of Cy5.5 measured in both groups were did not exceed the general background (Additional file 2: Figure S2B and C).

We subsequently performed an intra parenchymal injection of Cy5.5-AuNPs targeting the presumptive somatosensory cortex of mice. Due to the small volume of Cy5.5-AuNPs that we delivered in brains, the fluorescence levels of Cy5.5 were hardly detectable in living mice. However, we detected signals that were more robust when we scored FLI in isolated brains collected 5 days after the injection of particles (Additional file 3: Figure S3A). Signals were concentrated in spots localized in the presumptive somatosensory cortex (Additional file 3: Figure S3A). FLI of 500 µm thick coronal slabs further confirmed that highest Cy5.5 signal was deriving from the somatosensory cortex (Additional file 3: Figure S3B). AuNPs with a size of 20 nm absorb 530 nm wavelength and emit a wavelength that peaks at 610 nm, which is sufficiently intense to be detected by a fluorescence microscope [38]. Therefore, we performed serial imaging of adjacent sections, scoring the somatosensory cortical wall. We labelled 20 µm thick sections for NeuN and by confocal microscopy we observed the almost perfect co-localization of Cy5.5 signals with Au signals (Additional file 4: Figure S4A, B) in several cortical NeuN^+^ cells (Additional file 4: Figure S4C, D).

We next labelled parallel sections for the microglia/macrophages Iba1 to establish whether the injection procedures may alter the cortical morphology. We used Iba1 to label reactive microglia/macrophages, both associated to focal CNS injuries [39]. Iba1^+^ cells were scored in regions surrounding the site of the injection (ipsilateral cortex) as well as in the controlateral cortex, not receiving any manipulation (Additional file 3: Figure S3C, D). We observed a moderate to high gliosis featured by a significant increase of Iba1^+^ cells in the ipsilateral cortex (Additional file 3: Figure S3E). Therefore, we reasoned that the relative high abundance of Iba1^+^ Cy5.5-AuNPs^+^ cells in the ipsilateral cortex could mirror the activation of phagocytosis (Additional file 3: Figure S3F), a classical phenomenon that occurs in response to tissue damage [40].

**Fig. 3:**
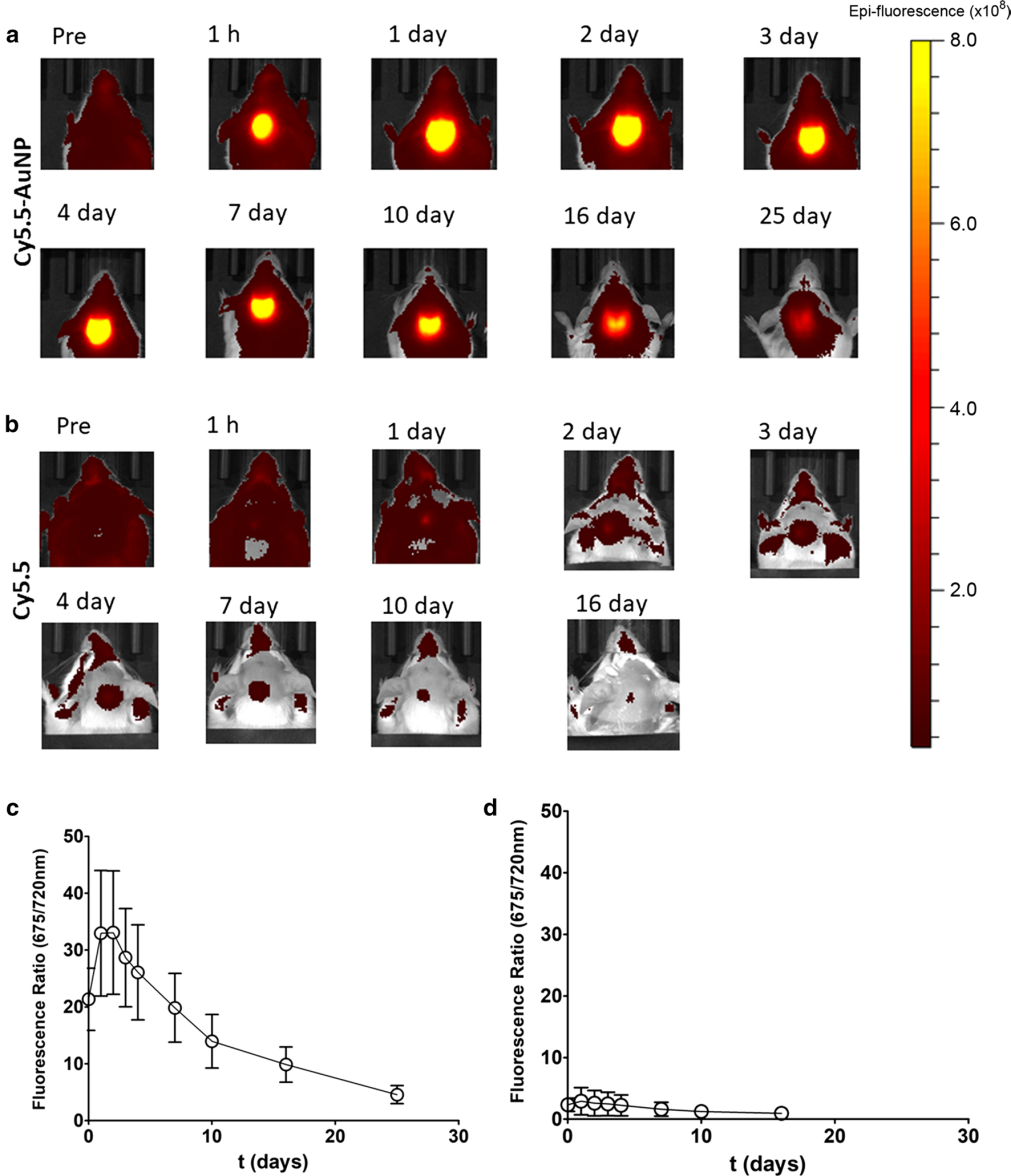
Longitudinal distribution of AuNPs in ICM-injected mice. **a** FLI imaging of a single animal receiving ICM injection of Cy5.5-AuNPs. Pictures were recorded at different time points (1 h, 1, 2, 3, 4, 7, 10, 16 and 25 days after the injection). FLI levels recorded in a control animal receiving ICM injection of unconjugated Cy5.5 are shown in **b**. The epi-fluorescence scale used in both experiments is shown on the right side of the panel. Plots in **c**, **d** show quantifications of FR levels (mean ± S.D.) in mice receiving Cy5.5-AuNPs (**c**) as well as in mice receiving soluble Cy5.5 (**d**), (n = 3 for each group)

We next performed ICM delivery of either Cy5.5-AuNPs or the unconjugated Cy5.5 [41]. Upon Cy5.5-AuNPs injections, we performed FLI analysis of Cy5.5 at different time points. Intense Cy5.5 fluorescence signals were detected in regions encompassing the 4th ventricle of mice injected with Cy5.5-AuNPs (Fig. 3a). Calculated FR levels peaked 1 day after the injection, although considerable high fluorescence signals were still detectable 25 days after the injection (Fig. 3c). On the other hand, scant fluorescence levels were observed in mice treated with the unconjugated Cy5.5 (Fig. 3b, d).

**Fig. 4:**
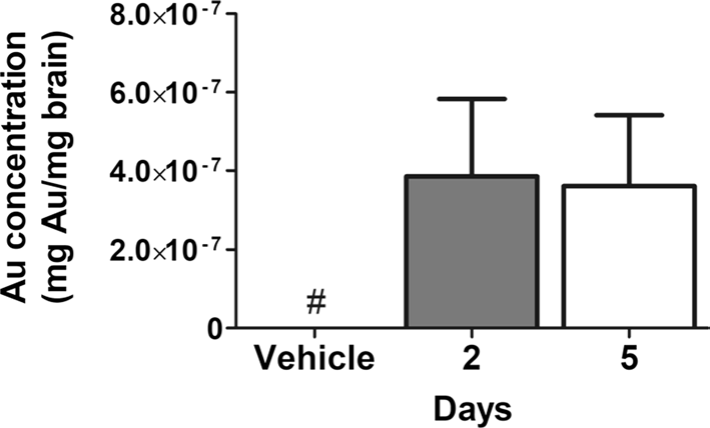
ICP-OES analysis of brain Au contents. Adult mice received ICM injections of Cy5.5-AuNPs or vehicle and brains were collected 2 and 5 days after the delivery (n = 3 for each group). Plot reports means (± S.D.) of the total amount of Au (mg) found in the brain homogenates (mg) at each time point

We next calculated Au concentrations in brain explants obtained from mice receiving ICM injection of Cy5.5-AuNPs. The Cy5.5-injected group was not included in this experiment since we reasoned that FR signals were close to background levels as shown in the previous experiment (Fig. 3d). Two and five days upon Cy5.5-AuNPs delivery, mice were perfused with saline to remove particles potentially retained in the blood circulation. We dissected each brain to obtain coronal slabs that spanned from the anterior bregma − 5.5 to the posterior bregma − 8.5. Explants were digested over a heating plate by adding a solution of HNO_3_/H_2_O_2_ 30% m/m (3/1) followed by *aqua regia*, and the amount of Au in each of them was detected by Inductively Coupled Plasma-Optical Emission Spectrometers (ICP-OES). Au was undetectable in vehicle-treated mice, while a significant amount of Au was detected in Cy5.5-AuNPs injected mice at both time points (Fig. 4).

### Ex-vivo localization of Cy5.5-AuNPs in ICM injected brains

**Fig. 5:**
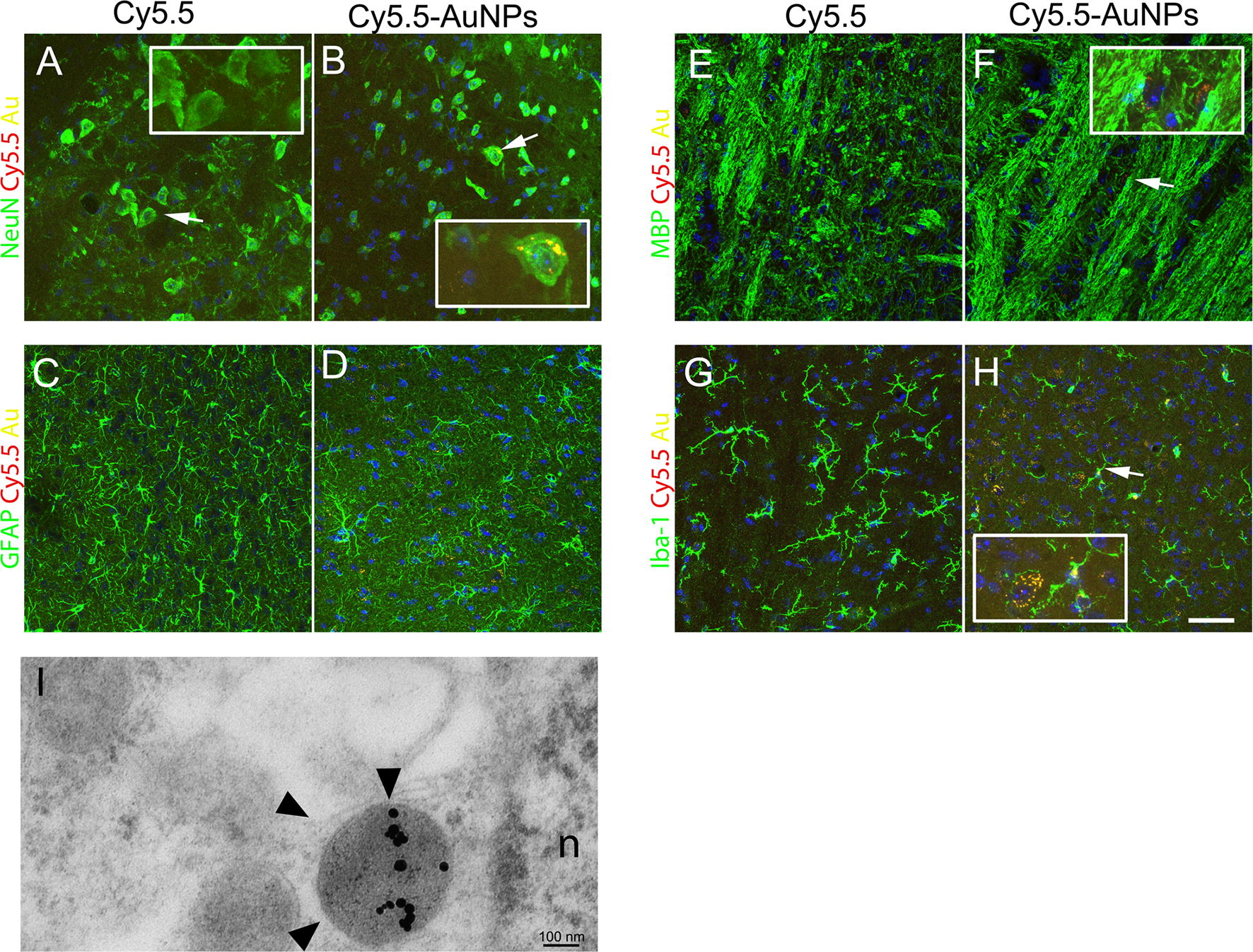
Distribution of Cy5.5-AuNPs in ICM-injected mice. Confocal images of sections (maximum projections) from the pons/medulla of mice sacrificed 30 days after ICM injections. Acquisitions were done to capture Cy5.5 and Au signals in control mice—i.e. receiving Cy5.5 (**A**, **C**, **E** and **G**)—and in mice receiving Cy5.5-AuNPs (**B**, **D**, **F** and **H**). **A**, **B** Triple labelling for NeuN, Cy5.5 and Au. Arrows indicate cells that are shown at high magnifications in insets. **C**, **D** Confocal images of adjacent sections labelled for Cy5.5, Au and GFAP. **E**, **F** Triple labelling for MBP, Cy5.5 and Au. Arrow in **F** indicates a region that is shown at high magnification in the inset. **G**, **H** Confocal images of sections labelled for Iba1, Cy5.5 and Au. The arrow in **H** indicates one Iba1^+^ cells that is shown at high magnification in the inset (n = 3 for each group). **I** Au nanoparticles localized in vacuoles of parenchymal cells of brains sampled at day 15. Arrows indicate several Au particles in a putative lysosome of a cell (n = 2). Scale bar 50 µm for **A**–**H**, 0.1 µm for **I**

**Fig. 6:**
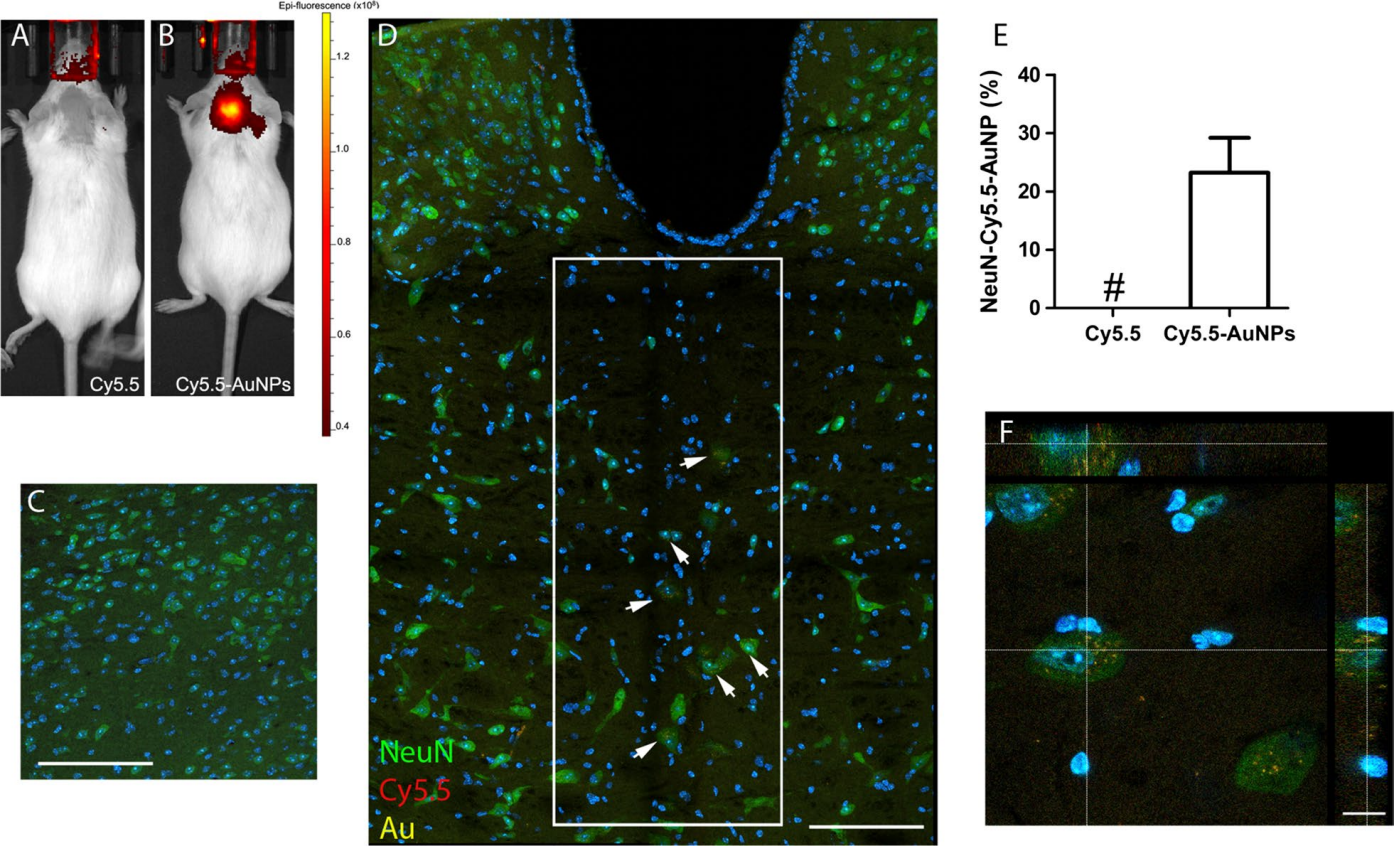
Distribution of Cy5.5-AuNPs^+^NeuN^+^ in ICM-injected mice. **A**, **B** FLI scans in a control mouse (Cy5.5) and in a mouse receiving Cy5.5-AuNPs both sacrificed 15 days after the injection. The Epifluorescence scale is plotted on the right side of **A**. Representative confocal scans for NeuN, Cy5.5 and Au in the pons/medulla of these mince are shown in **C**, **D** (n = 3 for each group). **D** Is a confocal image obtained stitching six independent pictures. Percentages of NeuN^+^Cy5.5^+^Au^+^ were established in mice receiving Cy5.5-AuNPs scoring cells in the superimposed ROI (200 × 500 µm) and data are plotted in **E**. Arrows of **D** show NeuN^+^Cy5.5^+^Au^+^ cells. **F** A confocal cross section of a NeuN^+^ cells expressing Au and Cy5.5. Scale bar 100 µm for **C**, **D** and 10 µm for **F**

We next investigated the uptake of Cy5.5-AuNPs in fixed brains sections, sampling brains from mice sacrificed 30 days after ICM delivery of Cy5.5-AuNPs. Using confocal microscope, we acquired Cy5.5 and Au fluorescence in order to increase the signal to noise ratio, the signal specificity, and to provide a better estimation of Cy5.5-AuNPs distribution [38]. We scored scattered NeuN^+^ cells in the presumptive medial vestibular nucleus that were positive for Cy5.5-AuNPs (Fig. 5A, B). Conversely, sections labelled for GFAP (Fig. 5C, D) and for the myelin marker MBP (Fig. 5E, F) showed very few, if any, cells containing Cy5.5-AuNPs. We next labelled sections for Iba1 and we observed some putative microglia displaying the ramified cell morphology of *bona fide* resting cells that were positive for Cy5.5-AuNPs (Fig. 5G, H). Control brains derived from mice injected with soluble Cy5.5 did not show any detectable signal for Cy5.5 and Au (Fig. 5A, C, E and G). We next assessed the localization of Cy5.5-AuNPs in the CNS by TEM, sampling sections in a region encompassing the 4th ventricles. Cy5.5-AuNPs were observed in few ependymal cells (not shown), while we observed particles in parenchymal cells. Nanoparticles were detected in some electron dense vacuoles, possibly belonging to lysosomal compartment (Fig. 5I). We next calculated percentages of NeuN^+^ Cy5.5^+^Au^+^ cells in the pons/medulla of ICM-injected mice that we sacrificed at day 15 (Fig. 6A, C). Control mice injected with unconjugated Cy5.5 displayed signals that were undistinguishable from background levels (Fig. 6A, C). On the other hand, we observed intense Cy5.5 levels in mice receiving particles (Fig. 6B). We next went to the tissue scoring Au and Cy5.5 in NeuN-expressing cells located in the pons/medulla, a region placed beneath the 4th ventricles (boxed area of Fig. 6D). Around 23% of NeuN^+^ cells showed Au and Cy5.5 signals in the cytoplasm (Fig. 6E, F). Of note, some triple positive cells were relatively far from the ventricular lining, suggesting that AuNPs can spread in the CNS parenchyma (arrows in Fig. 6D).

## Discussion

A considerable number of putative drugs lacks the ability to penetrate the BBB, and for this limitation none of them were further pursued as therapeutics. The functionalization of nanovectors with these molecules could, in principle, overcome such limitation, allowing their diffusion in diseased brains. Thus, the physiological barrier represented by the BBB could be crossed by nanoparticle-supported drugs able then to interact with their targets. However, after an initial enthusiasm for this approach, it became clear that the general efficiency of nanovectors to cross the BBB was modest [17–21]. In this context, AuNPs have attracted attention for their safety and biophysical characteristics [42]. The direct injection of AuNPs in the CNS parenchyma is attractive, although our data indicate that such procedure may generate tissue damage and microglia activation. Intranasal administration of AuNPs, conversely, leads to their uptake in different brain regions and is a promising method for the BBB passage of nanoparticles, although the regional distribution in the brain of molecules may vary from substance to substance, and additional studies are needed to validate this approach [43]. Repeated intravenous injections of nanoparticles, often using high dosages to achieve BBB permeation, could induce their accumulation in the liver causing inflammation and apoptosis and may alter neuronal functions [41, 44]. A recent study, showing in vivo instability of coated AuNPs, adds a further layer of complexity, suggesting that using AuNPs to deliver molecules as well as their tracking in living animals may be influenced by a certain degree of molecular instability and/or cell metabolism [45]. Indeed, fluorochrome-coated AuNPs are processed by lysosomes in the liver, and molecules tagging AuNPs can be cleaved by proteolysis [45]. These experimental observations should be considered in the interpretation of distribution studies after systemic administration of AuNPs.

For these reasons, we attempted to deliver Cy5.5-AuNPs directly in the brain using a single intrathecal injection. This approach has been extensively used in the past to deliver lentiviral particles and drugs [30]. ICM injections are well characterized for such vectors, but little is known regarding their use to deliver AuNPs. Our experimental evidence shows that this route of administration does not induce signs of distress in mice. It was surprising to observe, using in vivo optical imaging, that mice receiving Cy5.5-AuNPs showed retention of particles in the brain for more than 20 days. Furthermore, in this experimental setting we observed that the fluorescence emitted by both Cy5.5 and Au in brain sections were perfectly overlapped, suggesting a high stability of the Cy5.5 tag grafted on Au surfaces. These results suggest that functionalized AuNPs entering the CNS escape the degradation and are more stable in the CNS than in other organs, such as the liver [45]. The diffusion of Cy5.5-AuNPs in the CNS parenchyma was surprisingly wide, and some neurons positive for them localized several hundreds of microns away from the ventricular cavity. The mechanism of diffusion of such particles is still unknown, but we could speculate that these such small particles can passively diffuse in the extracellular matrix before being up taken by endocytosis. However, double staining of Cy5.5-AuNPs and markers of neurons, microglia and astrocytes, revealed that AuNPs uptake was prominent in neurons, while only few microglia efficiently incorporate such particles. The high stability of Cy5.5-AuNPs in the brain can be due to their rapid uptake by cells, that prevents their removal by convective lymphatic fluxes of the CSF [46]. In addition, PEG functionalization is helping to prevent their uptake by peri-vascular macrophage, thus increasing the half-life of Cy5.5-AuNPs. Thus, our study clarifies the routes and the mechanisms of functionalized AuNPs distribution, kinetic and stability after entering the brain. We observed a relevant AuNPs uptake in neurons and microglia far from the CM. We also observed their uptake in intracellular compartments (mainly lysosomes), suggesting a movement of Cy5.5-AuNPs through membrane compartments.

## Conclusion

We have demonstrated that Cy5.5-AuNPs are highly stable upon their entry into the CNS, probably due to their resistance to cellular metabolism. We have shown that AuNPs are efficiently taken up by neurons and concentrate into intracellular compartments. In principle, their long stability and their ability to target intracellular vesicles in neurons could be exploited in disorders showing disrupted lysosomal homeostasis in neurons, such as the wide family of Lysosomal Storage Diseases [47]. AuNPs could be also exploited in brain cancer radiotherapy, because PEG-coated AuNPs can accumulate in brain tumors and can be efficiently used for increasing radiotherapy efficiency [8]. Thus, our experimental evidence showing a long-lasting PEG-AuNPs accumulation in neurons upon intrathecal injection suggests that this route of administration could be potentially used for radiotherapy enhancing agents in CNS tumors as well as in Lysosomal Storage Diseases.

## Methods

### Synthesis of PEG-functionalized AuNPs

Synthesis of PEG-functionalized AuNPs was performed following our published methods [32]. Cyanine 5.5 (Cy5.5) NHS ester was purchased from Lumiprobe (Hunt Valley, Maryland 21030, USA) and stored in the dark at − 20 °C. All glassware used for AuNP synthesis was cleaned with aqua regia (HCl(37%)/HNO_3_(65%) 3/1). A water mixture of sodium citrate (9 ml, 2%), HAuCl_4_·_3_H_2_O (7.0 ml, 10 mM) and AgNO_3_ (420 μl, 0.1%) was stirred at room temperature for 6 min and added to 250 ml of boiling water. The mixture was stirred (750 rpm) for 1 h at 100 °C, then the seed solution was allowed to cool to room temperature. Glycerol (5 ml) was added and the suspension was stirred for 15 min. A second aliquot of sodium citrate (10 ml, 1%), HAuCl_4_ (7.5 ml, 10 mM) and AgNO_3_ (426 μl, 0.1%) was separately stirred for 6 min and then added to the colloidal solution, followed immediately by a solution of hydroquinone (8 ml, 1%). The resulting solution was allowed to age, stirring at 750 rpm for 1 h at room temperature. The reddish solution of citrate-capped AuNPs was directly used without purification to functionalize the gold surface. A mixture of CH_3_O-PEG_5000_-SH/NH_2_-PEG_5000_-SH 9/1 (30 mg, Rapp Polymer GmbH) was dissolved in 5 ml of water and NaOH (8 mg) and added to the mixture containing AuNPs while argon was bubbled in the solution (for 5 min). The reaction mixture was stirred for 48 h at room temperature. PEG-AuNPs were then concentrated and purified by mean of Amicon centrifugal Filter units to a final volume of 11 ml.

### Synthesis of Cy5.5-AuNPs

Five ml of PEG-functionalized AuNPs (10% PEG-NH_2_), in milliQ water, were filtered by centrifugal filtration (10,000 MWCO, Vivaspin filters) to remove the solvent, and were re-suspended in borate buffer at pH 8. To this solution, Cy5.5-NHS ester (450 µg, 0.00063 mmol, Lumiprobe-Hunt Valley, Maryland 21030, USA) dissolved in DMSO (500 µl) was added at room temperature. We adjusted pH to 8.5 by adding NaOH 0.2 N, and the reaction mixture was stirred at room temperature in the dark for ca. 18 h. Then, the solvent was removed by centrifugal filtration (10,000 MWCO, Vivaspin filters) and Cy5.5-AuNPs were washed with tBuOH/H_2_O 1:1 until the collected washing solution was colorless (the absence of Cy5.5 adsorption was checked through UV–Vis spectroscopy). Finally, Cy5.5-AuNPs were washed with milliQ water to remove tBuOH traces, were dissolved in 5 ml milliQ water and stored at 4 °C.

### Characterization of Cy5.5-AuNPs

Dynamic light scattering (DLS) was employed to measure hydrodynamic diameter and Zeta-potential, determined by using a 90 Plus Particle Size Analyzer from Brookhaven Instrument Corporation (Holtsville, NY) operating at 15 mW of a solid-state laser (λ = 661 nm), using a scattering angle of 90°, equipped with an AQ-809 electrode, operating at applied voltage of 120 V. DLS samples were prepared by filtration with a 0.45 μm cellulose acetate syringe filter before loading into the cuvette, in order to remove large interfering particulate matter. Each sample was allowed to equilibrate for 3 min prior to start the measurement. Three to ten independent measurements of 60 s duration were performed, at 25 °C. The hydrodynamic diameter calculation was performed using Mie theory. The absolute viscosity and refractive index values of the medium were respectively set to 0.911 cP and 1.334. The Zeta-potential was automatically calculated from electrophoretic mobility based on the Smoluchowski theory. A viscosity of 0.891 cP, a dielectric constant of 78.6, and a Henry function of 1.5 were used for the calculations. UV/Vis spectra were recorded on an Agilent 8453 instrument by using a disposable cuvette with 1 cm optical path length for the measurements. Transmission electron microscopy micrographs have been collected using a TEM-Zeiss LIBRA 200FE instrument, equipped with: 200 kV FEG, in column second-generation omega filter for energy selective spectroscopy (EELS) and imaging (ESI), HAADF STEM facility, EDS probe for chemical analysis, integrated tomographic HW and SW systems. TEM specimens were prepared by dropping an aqueous solution of AuNPs onto a carbon-coated copper grid (300 mesh) and evaporating the solvent. The particle size distribution was estimated by using ITEM-TEM Imaging platform—Olympus Soft Imaging Solutions. The number of measured nanoparticles for each sample resulted to be around 250. Au concentration was determined via Inductively Coupled Plasma-Optical Emission Spectrometers (ICP-OES; iCAP 6300 Duo, Thermofisher). Samples (1 ml each) were digested in a glass vial over a heating plate with *aqua regia* (2 ml), repeating the treatment for four times. The explant samples were digested over a heating plate with a solution of HNO_3_/H_2_O_2_ 30% m/m (3/1, 2 ml) for two times, followed by *aqua regia* (2 ml) for three times. The dry residues were dissolved in a 0.5 M HCl aqueous solution and diluted. The limit of detection (lod) calculated for gold was 0.01 ppm.

### Nanoparticle administration in vivo

Mice were maintained under pathogen-free conditions at San Raffaele Hospital mouse facility (Milan, Italy). All efforts were made to minimize animal suffering and to reduce the number of mice used in accordance with the European Communities Council Directive of 24 November 1986 (86/609/EEC). Five weeks CD1 (Charles Rivers) females were given an injection of anesthetic (2,2,2-tribromoethanol, 10 mg/ml; 1/27 of body weight), then 10 µl of Cy 5.5-AuNPs (0.11 µg/µl) were injected within the cisterna magna of mice using a 27-gauge stainless steel needle curved (40°) at 3.5 mm from the tip, so that it was J-shaped [48]. IV delivery was obtained injecting mice in the tail vein with 200 µl of Cy5.5-AuNPs (0.11 µg/µl). Intra parenchymal injection was obtained by delivering 1 μl of Cy 5.5-AuNPs (1.1 µg/µl) in the brain by a single stereotaxic injection at the following coordinates: A − 0.5, 0; L, + 1.2; and D, − 0.8 [49]. Control mice received injections of soluble Cy5.5 or vehicle (sterile saline). To calculate the appropriate amount of Cy5.5 to be delivered into the cisterna magna, we compared curves of fluorescence from serial dilutions (dilution factor = 10) of Cy5.5 and Cy5.5-AuNPs, using the IVIS SpectrumCT System. Control mice received Cy5.5 at the concentration that produced the same fluorescence level of the Cy5.5-AuNPs used in our experiments. At the sacrifice (2, 5, 15 and 30 days after the delivery of particles), mice were given an overdose of anesthetic drugs and were transcardially perfused with saline followed by 80/100 ml 4% paraformaldehyde in PBS 1×, pH 7.2 (Sigma). Brains were post fixed in the same solution for 12 h at + 4 °C. Tissues were cryoprotected in PBS/30% Sucrose (Sigma), embedded in OCT inclusion media and stored at − 80 °C before processing. The metal content in brain explants was determined by ICP-OES. Mice were perfused with sterile saline and brains homogenized in PBS solutions. Sample were digested in a glass vial over a heating plate with *aqua regia* according our published methods [32]. The dry residuals were dissolved in a HCl aqueous solution 0.5 M and properly diluted.

### In vivo imaging

Mice images were acquired by placing the animals at 37 °C under gaseous anesthesia (2–3% isoflurane and 1 l/min oxygen). FLI was performed by IVIS Spectrum-CT System (Perkin Elmer), equipped with a low noise, back-thinned, back-illuminated CCD camera cooled at − 90 °C and a with quantum efficiency in the visible range > 85%. Images were obtained using the following settings: exposure time = auto, binning = 8, f = 2 and a field of view equal to 13 cm (field C); when needed spectral unmixing, FLI was obtained using the following excitation/emission filters: 640/680, 640/700, 640/720, 640/740, 675/720, 675/740, 675/760 nm. Acquisitions were done before and after the injection of particles with the following time sampling: 1 h, and 1, 2, 3, 4, 7, 10, 16, 25 days after the delivery of Cy5.5-AuNPs. Analyses were done in a region of interest (ROI) overlapping the brain, measuring the radiant efficiency within this ROI using images acquired with the 675/720 filters. The FR(t_i_) was calculated on ROI sampled at different time points (t_i_) and normalized on images acquired before injection or particles (t_0_) to quantify the magnitude of the fluorescence signal: FR(t_i_) = [ROI(t_i_))/(ROI(t_0_)]. Spectral un-mixing of the FLI data was performed on selected images in order to show the specificity of the fluorescence signal over the tissue auto-fluorescence. All the images were acquired and analyzed using Living Image 4.5 (Perkin Elmer).

### Immunofluorescence and electron microscopy

Immunofluorescence and electron microscopy have been done according the following methods [49–51]. Brains were sagittal or coronal sectioned (20 µm) and one section every 250 µm was used for immunofluorescence. Slides were washed in PBS 1× and incubated with the following blocking buffer: PBS 1×, BSA 1 mg/ml, FBS 10%, Triton 0.1% (Sigma). Primary antibodies were diluted in the same buffer and incubated on section for 12 h at + 4 °C. Secondary antibodies (Alexa-flour conjugated) were incubated according to manufacturer’ instructions, then sections were cover slipped with Dako mounting mix. The following antibodies were used: mouse α-NeuN (Millipore, 1:800); rabbit α-MBP (Millipore) 1: 500; rabbit α-Iba1 (Wako) 1:400; rabbit α-GFAP (Dako) 1:1000. Secondary, Alexafluor 488-conjugated antibodies were used according to manufactures’ instructions. Acquisitions were performed with a Leica SP8 confocal microscopy equipped with a 40× objective and with super-sensitive HyD detectors. Fluorescence was recorded as square 8-bit images (1024 × 1024 pixels) and stored as separate image stacks for each channel. Alignment of images to obtain largest field of view of coronal sections was done by automatic stitching of stack images using the Leica (Las-X, Leica). Acquisition of fluorescence from Au was done according published methods [38]. We used an excitation wavelength of 532 nm, while signals were acquired in the 594–640 nm window. Images showed the maximal projections of Z-stacks acquired with a 0.8 µm step or cross sections of selected cells acquired with 0.3 µm step. Images were pseudo-colored using Las-X software. For EM analysis, brains were post-fixed in 0.12 M phosphate buffer supplemented with 2% glutaraldehyde, and further sectioned to get a region encompassing the 4th ventricle and the cerebellum. These parts were further post-fixed with osmium tetroxide and embedded in Epon (Fluka, Buchs, Switzerland). Ultrathin sectioning of the CNS allowed the generation of 70 nm sections that were imaged by a transmission electron microscope (LEO 912AB). In vitro quantification of Cy5.5-AuNPs fluorescence were performed using the FLI (FLI) acquired with IVIS SpectrumCT System scanner (Perkin Elmer).

### Primary hippocampal cultures and sytox determination

Neurons were obtained from the hippocampus of CD1 (Charles Rivers) mice at the embryonic stage of E17.5. Hippocampi were mechanically dissociated in cold HBSS supplemented with 0.6% glucose, 5 mM HEPES (pH 7.4) (Sigma Aldrich). Cells were suspended in culture medium containing Neurobasal Medium (Thermo Fisher Scientific) supplemented with: N2 (Thermo Fisher Scientific), B27 (GIBCO), 5 mM HEPES (pH 7.4), 0.6% glucose (Sigma Aldrich) and 0.5% glutamine (Thermo Fisher Scientific) in the absence of antimitotic and antibiotic drugs. Fifteen days after plating neurons were incubated with increasing amounts of Cy 5.5-AuNPs (1, 0.1, 0.01 and 0.001 mg/ml) for 24 h. Percentages of dying cells were estimated labelling cells with Sytox and Hoechst (Molecular Probes). Automated imaging was performed using the ArrayScan^®^ (ThermoFisher) equipped with a 40× air objective. More than 300 fields per conditions have been assayed for the statistical evaluation of cell death.

### Statistics

Data are expressed with the mean ± standard error (± S.D.). Normality of dataset was assessed in each experiment by applying either Kolmogorov–Smirnov test (with Dallas–Wilkinson–Lille for P value) or Skewness test. Comparisons were done using: unpaired t-test, one-way analysis of variance (ANOVA), followed by Tukey post hoc test. Statistical tests were carried out using PRISM5.01 (GraphPad Software).

## Additional files

**Additional file 1: Figure S1.** Synthetic route for Cy5.5-AuNPs. General scheme for the synthesis of surface-engineered Cy5.5-AuNPs. PEG-AuNPs are synthesized using sodium citrate as reducing agent and preliminary capping ligand. Purified PEG-AuNPs have been functionalized by forming a stable amide bond between PEG-NH_2_ and NHS activated chromophore Cy5.5.

**Additional file 2: Figure S2.** Cy5.5-AuNPs in tail vein-injected mice. Panels A shows the FLI analysis of mice receiving a single injection of Cy5.5-AuNps in the tail vein that were sacrificed 5 days after the injection. Epifluorescence scale is plotted on the left side of the panel A. A representative confocal scan of the cerebral cortex showing NeuN and Cy5.5 in mice injected with the vehicle and in mice injected with Cy5.5-AuNPs are shown in panels B and C, respectively (n = 3 for each group). Scale bar 100 µm.

**Additional file 3: Figure S3.** Cy5.5-AuNPs in intra parenchymal injected mice. Panel A shows FLI analysis in brains receiving a single intra parenchymal injection of Cy5.5-AuNPs. Epi-fluorescence scale is plotted on the right side of the panel. Arrow in panel A indicates one of these brains that was subsequently sectioned in coronal slabs and further assayed for FLI FLI analysis (B). Epi fluorescence scale is plotted on the right side of the panel. Panels C and D show confocal scans of the cerebral cortex from a Cy5.5-AuNPs-injected mouse labelled for Iba1, Cy5.5 and Au. Iba1^+^ cells were scored in both the contralateral (C) and the ipsilateral cerebral cortex (D). Percentages (± S.D.) of Iba1^+^ cells are shown in the histogram of panel E (n = 3). Arrow in panel D indicates a single Iba1^+^ cell that is shown at high magnification in the confocal cross section of panel F. Scale bar 100 µm. * p < 0.05 unpaired t test.

**Additional file 4: Figure S4.** Co-localization of Au, Cy5.5 and NeuN in cortical neurons. Coronal sections from mice receiving a single intra-parenchymal injection of Cy5.5-AuNPs were labelled for NeuN (C) and submitted to confocal imaging for Au (A) and Cy5.5 (B). To maximize the probability to find triple positive cells we did the imaging in the cortical wall in a region that was adjacent the site of injection. Merge panel in D show co-localization of Cy5.5 and Au in NeuN^+^ cells (n = 3). Scale bar 30 µm.

## Abbreviations

AuNPs: gold nanoparticles
CNS: central nervous system
BBB: blood brain barrier
PEG: polyethylene glycol
CFS: cerebrospinal fluid
ICM: intra cisterna magna
FLI: fluorescence imaging
TEM: transmission electron microscopy
FR: fluorescence ratio
ICP-OES: Inductively Coupled Plasma-Optical Emission Spectrometers
DLS: dynamic light scattering
ROI: region of interest
