# Supplementary figures and images for: Intracisternal delivery of PEG-coated gold nanoparticles results in high brain penetrance and long-lasting stability
Source: J Nanobiotechnology. 2019 Apr 3;17:49. doi: 10.1186/s12951-019-0481-3 (PMC6448280; doi:10.1186/s12951-019-0481-3)

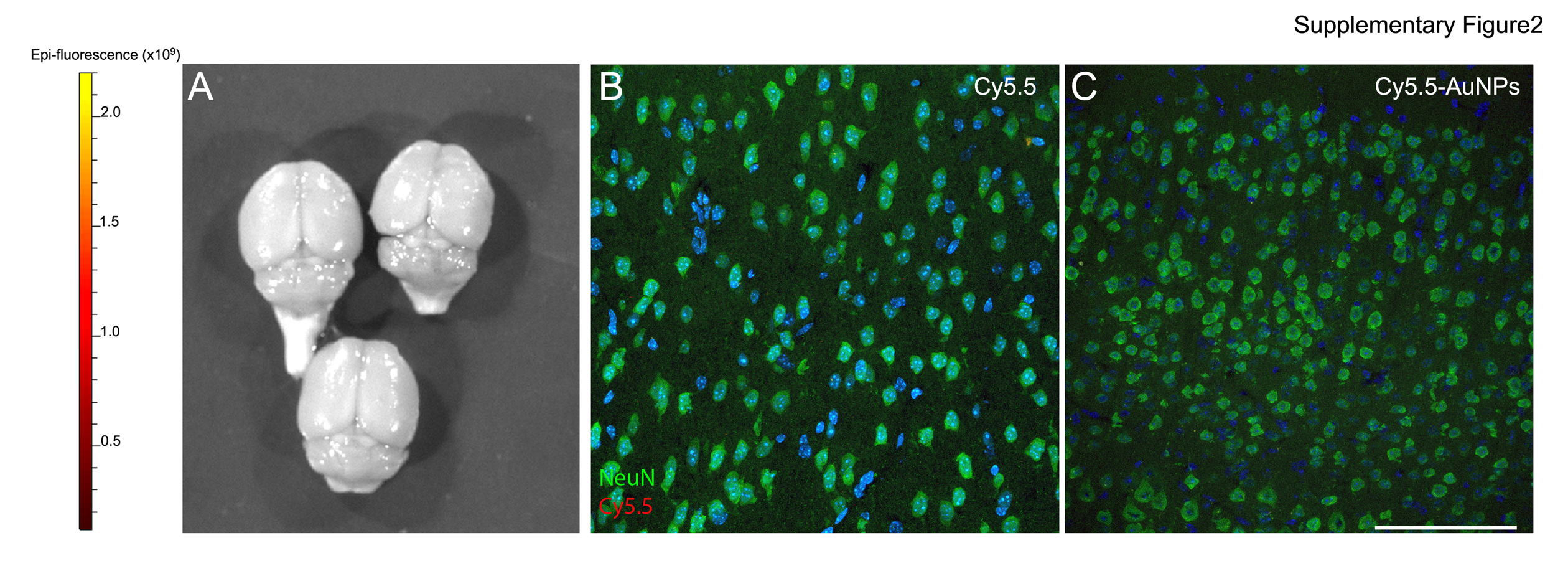

Supplement: Supplementary file 2 — Additional file 2: Figure S2. Cy5.5-AuNPs in tail vein-injected mice. Panels A shows the FLI analysis of mice receiving a single injection of Cy5.5-AuNps in the tail vein that were sacrificed 5 days after the injection. Epifluorescence scale is plotted on the left side of the panel A. A representative confocal scan of the cerebral cortex showing NeuN and Cy5.5 in mice injected with the vehicle and in mice injected with Cy5.5-AuNPs are shown in panels B and C, respectively (n = 3 for each group). Scale bar 100 µm. [file 12951_2019_481_MOESM2_ESM.tif]

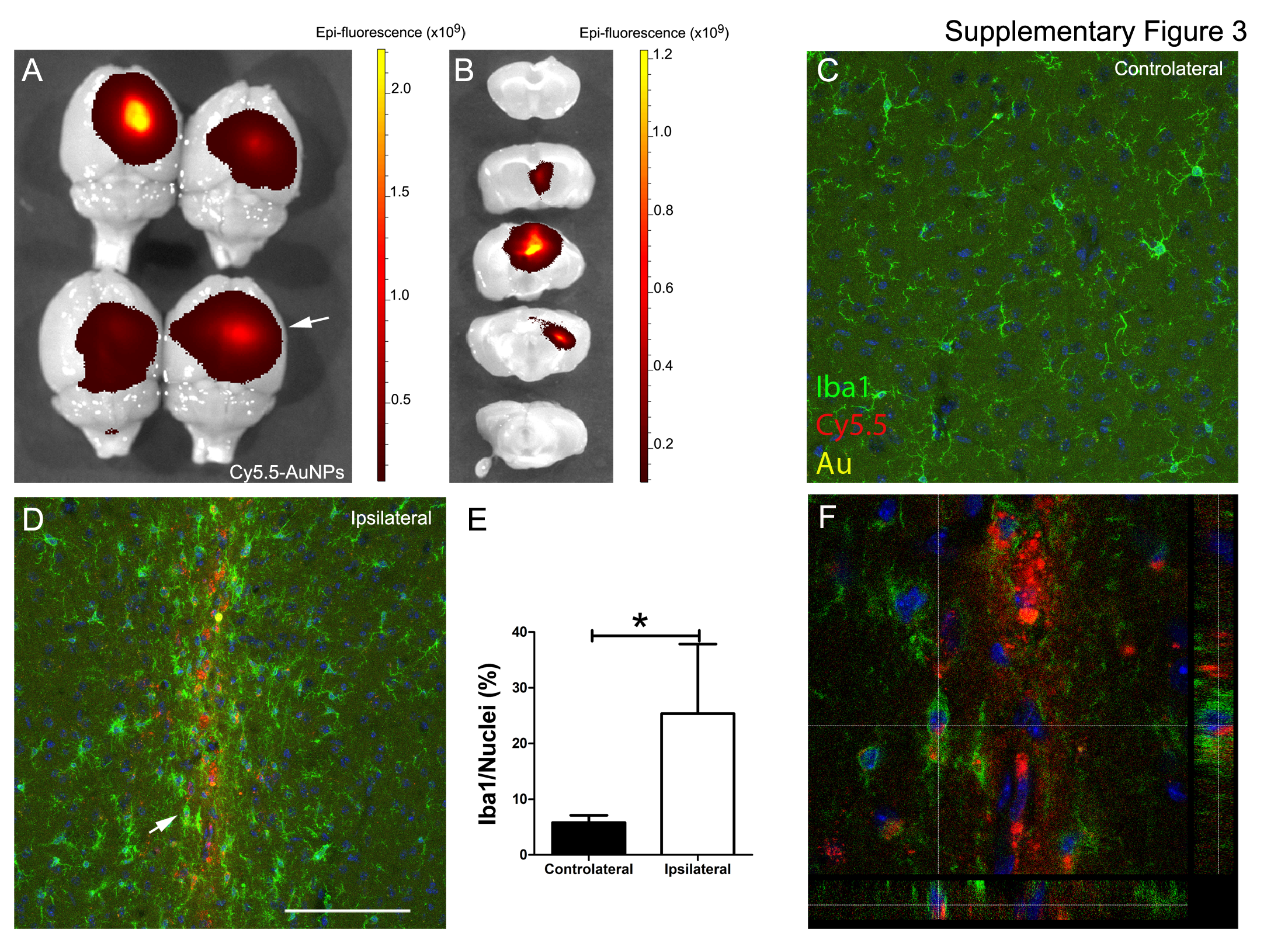

Supplement: Supplementary file 3 — Additional file 3: Figure S3. Cy5.5-AuNPs in intra parenchymal injected mice. Panel A shows FLI analysis in brains receiving a single intra parenchymal injection of Cy5.5-AuNPs. Epi-fluorescence scale is plotted on the right side of the panel. Arrow in panel A indicates one of these brains that was subsequently sectioned in coronal slabs and further assayed for FLI FLI analysis (B). Epi fluorescence scale is plotted on the right side of the panel. Panels C and D show confocal scans of the cerebral cortex from a Cy5.5-AuNPs-injected mouse labelled for Iba1, Cy5.5 and Au. Iba1+ cells were scored in both the contralateral (C) and the ipsilateral cerebral cortex (D). Percentages (± S.D.) of Iba1+ cells are shown in the histogram of panel E (n = 3). Arrow in panel D indicates a single Iba1+ cell that is shown at high magnification in the confocal cross section of panel F. Scale bar 100 µm. * p < 0.05 unpaired t test. [file 12951_2019_481_MOESM3_ESM.tif]

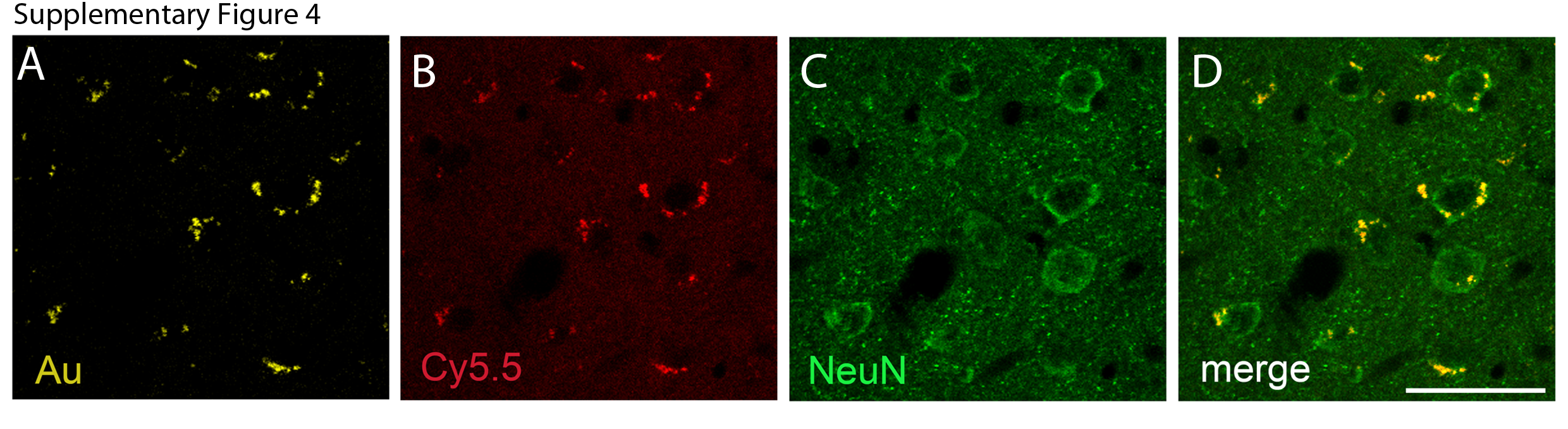

Supplement: Supplementary file 4 — Additional file 4: Figure S4. Co-localization of Au, Cy5.5 and NeuN in cortical neurons. Coronal sections from mice receiving a single intra-parenchymal injection of Cy5.5-AuNPs were labelled for NeuN (C) and submitted to confocal imaging for Au (A) and Cy5.5 (B). To maximize the probability to find triple positive cells we did the imaging in the cortical wall in a region that was adjacent the site of injection. Merge panel in D show co-localization of Cy5.5 and Au in NeuN+ cells (n = 3). Scale bar 30 µm. [file 12951_2019_481_MOESM4_ESM.tif]
